# Association of lifestyle habits and academic achievement in Norwegian adolescents: a cross-sectional study

**DOI:** 10.1186/1471-2458-14-829

**Published:** 2014-08-11

**Authors:** Tonje H Stea, Monica K Torstveit

**Affiliations:** Department of Public Health, Sport and Nutrition, University of Agder, Po Box 422, 4604 Kristiansand, Norway

**Keywords:** Adolescents, Academic achievement, Diet, Meal pattern, Physical activity, Smoking, Snuffing

## Abstract

**Background:**

While healthy lifestyle habits are generally assumed to be important for high academic achievement, there has been little research on this topic among adolescents. The aim of this study was therefore to examine the associations between several lifestyle habits and academic achievement in adolescent girls and boys.

**Methods:**

The study included 2,432 Norwegian adolescents, 15–17 years old. A self-report questionnaire was used to assess dietary-, physical activity-, smoking- and snuffing habits and academic achievement. Logistic regression models were adjusted for body mass index (BMI) and parental education.

**Results:**

In both girls and boys, high academic achievement was associated with a regular consumption of breakfast (AOR: 3.30 (2.45-4.45) and AOR: 1.76 (1.32-2.34), respectively) and lunch (AOR: 1.44 (1.08-1.93) and AOR: 1.43 (1.09-1.89), respectively), and in boys, with a regular consumption of dinner (AOR: 1.44 (1.16-1.79)) and a regular meal pattern in general (AOR: 1.50 (1.10 – 2.03)). In both girls and boys, high academic achievement was associated with a high intake of fruit and berries (AOR: 2.09 (1.51-2.88) and AOR: 1.47 (1.04-2.07), respectively), and in girls, with a high intake of vegetables (AOR: 1.82 (1.30-2.53)). In both girls and boys, high academic achievement was associated with a high leisure time physical activity level (AOR: 1.51 (1.10-2.08) and AOR: 1.39 (1.05-1.85), respectively) and use of active commuting (AOR: 1.51 (1.10-2.08) and AOR: 1.72 (1.26-2.35), respectively). In both girls and boys, high academic achievement was associated with a low intake of lemonade (AOR: 0.42 (0.27-0.64) and AOR: 0.67 (0.48-0.94), respectively), and in girls, with a low intake of sugar-sweetened soft drinks (AOR: 0.47 (0.35- 0.64)) and salty snacks (AOR: 0.63 (0.47-0.85)). Lastly, high academic achievement was inversely associated with smoking and snuffing in both girls (AOR: 0.18 (0.12-0.25) and AOR: 0.25 (0.17-0.37), respectively) and boys (AOR: 0.37 (0.25-0.54) and AOR: 0.51 (0.36-0.72), respectively).

**Conclusions:**

A regular meal pattern, an intake of healthy food items and being physically active were all associated with increased odds of high academic achievement, whereas the intake of unhealthy food and beverages, smoking cigarettes and snuffing were associated with decreased odds of high academic achievement in Norwegian adolescents.

## Background

Unhealthy lifestyle habits among children and adolescents is a major health concern, and some studies have also indicated a negative association between unhealthy lifestyle habits and decreased cognitive function and academic achievement [1–4]. A literature review concluded that skipping breakfast had a negative impact on academic achievement by adversely affecting cognition and absenteeism in both children and adolescents [5]. Although the evidence is not conclusive, studies have also demonstrated that the intake of a healthy breakfast is associated with an improved cognitive function and/or academic achievement in children and adolescents [6–8]. Whereas most of the previously published studies have focused on the effect of breakfast consumption, Kim and coworkers [9] reported a general association between the consumption of regular meals, including breakfast, lunch and dinner, and academic achievement in Korean children. Furthermore, a less frequent intake of unhealthy foods and not skipping meals was associated with a decreased odds of self-reported learning difficulties in Norwegian adolescents [10], while having a low overall diet quality was associated with a low academic score in Canadian children [11].

Literature reviews on physical activity and cognition have concluded that physical activity favorably affects cognitive functioning and ultimately an academic score [4, 12]. Fox and coworkers [13] reported that both physical activity and sports team participation were each independently associated with a higher academic achievement for adolescent girls, whereas only sports team participation was independently associated with a higher academic achievement for adolescent boys. Increasing the time and intensity of Physical Education has also been suggested to have a positive effect on cognitive performance and academic achievement among adolescents [14].

Only a few studies have examined both physical activity and dietary habits and the relationship with academic achievement in adolescents. A Spanish study reported that a high academic score was positively associated with both physical activity and fruit consumption in adolescent girls, but not in boys [15]. Edwards and coworkers [16] reported an association between higher math scores and positive nutrition behaviors, such as more milk and breakfast, less 100% fruit juice and sweetened beverages and increased physical activity and fitness.

Few studies have explored the relationship between active commuting and academic achievement. A study among Spanish adolescents, however, showed that active commuting to school was associated with better cognitive performance in girls, but not in boys [17].

A recently published longitudinal study including four time points (ages 12, 14, 17 and 19–27) showed that smoking behaviors at ages 12 and 14 predicted a lower academic achievement at later time points, and a lower academic achievement in adolescence predicted a higher likelihood of engaging in smoking behaviors [18]. Another study suggested that adolescents with high levels of academic achievement were less likely to smoke [19]. To the best of our knowledge, no studies have focused on the association between snuffing (use of smokeless tobacco) and academic achievement in school.

In conclusion, few studies have examined the association between several health-related behaviors and academic achievement in adolescents. Also, previous studies have described a need for more studies addressing multiple lifestyle factors to better understand the relationships between lifestyle and academic performance and identify differences between genders [15].

Thus, the purpose of this cross-sectional study was to examine the association between self-reported meal pattern, food and beverage intake, physical activity habits, smoking and snuffing habits and academic achievement in Norwegian boys and girls.

## Methods

### Study design and participants

This cross-sectional study is part of a large school-based, cluster-randomized intervention study, “Active and Healthy Youth.” The target group was the total population of students in the 1st grade at high schools in the south of Norway. In agreement with school boards/school principals, a total of 17 out of 23 schools (73.9%) decided to participate in the study. The main reason why six schools did not want to participate was due to a lack of time and a participation in other public health projects. A total of 2,619 out of 2,653 eligible students agreed to participate (98.7%) and responded by filling in a questionnaire. The data collection was conducted in classrooms or auditoriums, with at least one member of the project team present to inform about the project and answer possible questions. Before the questionnaires were handed out, the participants were informed that their responses would be treated as anonymous and that it was voluntary to participate. The students were given both oral and written instructions on how to fill out the questionnaire, and the students used approximately 30 minutes to answer the questionnaire. During the data analyses, a total of 187 students were excluded, as they did not meet the age requirements for participation in the study (15–17 years of age). Thus, the data analyses in the present study were based on a total number of 2,432 participants, 1258 girls (51%) and 1187 boys (49%). The Regional Committee for Medical Research Ethics approved the study protocol, and written consent was obtained from the students prior to participation in the present study. Although the Regional Committee for Medical Research Ethics did not require parental consent for adolescents aged 15 years and older in the present study, all students were encouraged to inform their parents about their participation and show them the information letter that described every detail of the study. Furthermore, the data collection was conducted during school time and principals, teachers and school nurses were given written information about the study.

### Instruments

The questionnaire included questions about gender, body weight and height, parental education, selected food and beverages, meal frequency, leisure time physical activity level, active commuting, smoking- and snuffing habits and academic score.

In order to test the reliability of the questions used in the present study, we conducted a test-retest study, including 143 adolescents between 15–17 years old. The results exhibited a good test-retest reliability, with an intra-class coefficient (ICC) ranging from 0.66 to 0.99.

Self-reported weight and height were used to calculate body mass index (BMI) (kg/m^2^). To estimate the prevalence of overweight and obesity, adolescent BMI categories were calculated using sex- and age-specific International Obesity Task Force (IOTF) cut-off points for defining overweight and obesity in children and adolescents aged 2–18 [20]. The ICC was 0.98 (95% CI 0.98-0.99) for weight and 0.99 (95% CI 0.98-0.99) for height.

The parental educational level was assessed with the question: “*What level of education do your parents have?*” The question had four response alternatives: (*i) elementary school, (ii) high school, (iii) college or university (≤3 years) and (iv) college or university (>3 years).* These response alternatives were then dichotomized into lower and higher education levels (lower = no college or university education; higher = having attended college or university). The ICC was 0.83 (95% CI 0.77-0.88) for maternal education and 0.80 (95% CI 0.72-0.85) for paternal education.

Academic achievement was assessed using grades from three core mandatory academic classes in high school, including Norwegian, English and Mathematics. The grade system in Norwegian High School is from 0 to 6, in which 6 is the best possible grade to obtain. Based on the self-reported school grades from these classes, mean school grades were calculated and used in the analyses. The ICC was 0.76 (95% CI 0.65-0.84) for achieved grades in Norwegian, 0.83 (95% CI 0.75-0.89) for achieved grades in English and 0.82 (95% CI 0.73-0.88) for achieved grades in Mathematics.

Meal frequency was assessed by questions such as: *“How often do you have breakfast each week*?” The same was asked for lunch, dinner and evening meals. Response alternatives ranged from never to seven days a week, and these were dichotomized into having meals fewer than seven times a week and having meals every day. Adolescents who were having these main meals every day were classified as regular breakfast-, lunch-, dinner- and evening meal consumers. These dichotomous variables were then combined to create a summary variable referred to as “all regular meals,” i.e. those eating all meals every day vs. those skipping meals, respectively. The ICC was 0.91 (95% CI 0.86-0.94) for breakfast, 0.78 (95% CI 0.68-0.85) for lunch, 0.68 (95% CI 0.55-0.79) for dinner and 0.89 (95% CI 0.83-0.93) for the evening meal.

Diet and beverage intake was assessed by asking: *“How often do you eat/drink….?”* All items had eight different response alternatives: *never, less than once a week, once a week, twice a week, . . . , 6 times a week, every day, several times every day*, and for the statistical analyses, they were scored 0, 0.5, 1, 2, . . . , 7, 10 times per week. Intakes of healthy food items, including fruits, berries and vegetables, were dichotomized to less than once a day and once a day or more. A consumption of once a day or more was seen as an acceptable frequency of consumption for these food items. Intakes of unhealthy food items and beverages, including lemonade, sugar-sweetened soft drinks, diet soft drinks, candy and salty snacks were dichotomized into three times a week or less and more than three times a week, in which the first mentioned category, was seen as an acceptable frequency of consumption. The ICC was 0.70 (95% CI 0.57-0.80) for fruit and berries, 0.73 (95% CI 0.61-0.82) for vegetables, 0.81 (95% CI 0.71-0.87) for lemonade, 0.85 (95% CI 0.77-0.90) for sugar-sweetened soft drinks, 0.75 (95% CI 0.64-0.83) for diet soft drinks, 0.80 (95% CI 0.70-0.86) for candy and 0.75 (95% CI 0.63-0.83) for salty snacks.

Leisure time physical activity level was assessed by asking: “*How many hours per week do you spend on doing sports/physical activity in a way that makes you breathless or sweat?”* The response alternatives were: “*0 hours, 1–2 hours, 3–4 hours, 5–7 hours, 8–10 hours and 11 hours or more.”* For the statistical analysis, the response alternatives were dichotomized into 0–4 hours per week and 5 or more hours per week of leisure time physical activity. The ICC was 0.91 (95% CI 0.87-0.93) for physical activity.

Information about active commuting was enquired about as follows: “*How do you usually commute to/from school?”* The response alternatives were: *“Walking, cycling, bus, car, MC/scooter, other alternatives (open alternatives).”* This variable was dichotomized into active commuting, which represented walking or cycling and non-active commuting. The ICC was 0.85 (95% CI 0.79-0.90) for commuting to school.

Smoking and snuffing habits were assessed by the question: “*Do you smoke/use snuff?”* The response alternatives were: *“Have never smoked/snuffed; have tried smoke/snuff, but not anymore, have smoked/snuffed regularly, but not anymore; smoking/snuffing, but not regularly* and *smoking/snuffing regularly and about__ cigarettes/day.”* For the statistical analysis, those who reported smoking or snuffing occasionally or daily were classified as being a smoker/snuffer. The ICC was 0.95 (95% CI 0.92-0.97) for smoking and 0.93 (95% CI 0.90-0.96) for snuffing.

### Statistical Analysis

ICC was used for test-retest reliability. Multiple logistic regressions were used to explore the association between academic achievement, meal pattern, intake of selected food items and drinks, leisure time physical activity level, active commuting, smoking and snuffing. The analyses were performed for girls and boys separately, and the dependent variable, academic achievement, was dichotomized into high and low grades. A mean school grade between 0 and 3 was characterized as low, whereas a mean school grade between 4 and 6 was characterized as high. All models were adjusted for both BMI and the educational level of the mother and father. Adjusted odds ratios (AORs) are presented with 95% confidence intervals (CIs), and a two-tailed p-value of <0.05 was considered to be statistically significant. All statistical analyses were performed using the SPSS statistical package version 19.0 (SPSS Inc., Chicago, IL, USA).

## Results

The mean age of the participating sample was 16.0 years (SD: 0.4 years), while the mean BMI was 21.7 (SD: 4.2) kg/m^2^ for the girls and 22.2 (SD: 3.7) kg/m^2^ for the boys.

Table 1 presents the meal pattern relative to academic achievement in adolescents. The results showed increased odds of high academic achievement in girls and boys who had a regular consumption of breakfast (AOR: 3.30 (2.45-4.45) and AOR: 1.76 (1.32-2.34), respectively) and lunch (AOR: 1.44 (1.08-1.93) and AOR: 1.43 (1.09-1.89), respectively) after adjustment for BMI and parental education. Increased odds of high academic achievement was also shown in boys who had a regular consumption of dinner (AOR: 1.44 (1.16-1.79)) and ate all four meals regularly (AOR: 1.50 (1.10 – 2.03)).

**Table 1:** **Adjusted odds ratio (AOR) and 95% CI for high academic achievement in relation to meal pattern in girls and boys**

	GIRLS			BOYS		
	Low academic achievement ^a^ n (%)	High academic achievement ^a^ n (%)	AOR ^b^(95% CI)	Low academic achievement ^a^ n (%)	High academic achievement ^a^ n (%)	AOR ^b^(95% CI)
**Regular breakfast** (7 times per week)	132 (40)	491 (66)	3.30 (2.45-4.45)*	217 (55)	424 (70)	1.76 (1.32-2.34)*
**Regular lunch** (7 times per week)	139 (42)	350 (47)	1.44 (1.08-1.93)*	174 (44)	333 (55)	1.43 (1.09-1.89)*
**Regular dinner** (7 times per week)	212 (64)	506 (68)	1.32 (0.98-1.78)	273 (69)	490 (81)	1.44 (1.16-1.79)*
**Regular supper** (7 times per week)	93 (28)	186 (25)	0.89 (0.64-1.24)	162 (41)	272 (45)	1.20 (0.91-1.59)
**Regular all four meals** (7 times per week)	43 (13)	112 (15)	1.19 (0.78-1.82)	87 (22)	175 (29)	1.43 (1.03-1.98)*

^a^A mean school grade between 0 and 3 was characterized as low, whereas a mean school grade between 4 and 6 was characterized as high.

^b^Adjusted for BMI and maternal and paternal education.

*p < 0.05.

Table 2 presents intake of selected food items and beverages relative to academic achievement in adolescents. Adjusted analyses showed increased odds of high academic achievement in girls and boys who had a high intake of fruit and berries (AOR: 2.09 (1.51-2.88) and AOR: 1.47 (1.04-2.07), respectively) and girls who had a high intake of vegetables (AOR: 1.82 (1.30-2.53)). Adjusted analyses revealed decreased odds of high academic achievement in girls and boys reporting a high intake of lemonade (AOR: 0.42 (0.27-0.64) and AOR: 0.67 (0.48-0.94), respectively) and girls reporting a high intake of sugar-sweetened soft drinks (AOR: 0.47 (0.35- 0.64)) and salty snacks (AOR: 0.63 (0.47-0.85)).

**Table 2:** **Adjusted odds ratio (AOR) and 95% CI for high academic achievement in relation to the intake of healthy and unhealthy food items in girls and boys**

	GIRLS			BOYS		
	Low academic achievement ^a^ n (%)	High academic achievement ^a^ n (%)	AOR ^b^(95% CI)	Low academic achievement ^a^ n (%)	High academic achievement ^a^ n (%)	AOR ^b^(95% CI)
**Vegetables** (≥7 times per week)	79 (24)	253 (34)	1.82 (1.30-2.53)*	71 (18)	133 (22)	1.33 (0.94-1.88)
**Fruit and berries** (≥7 times per week)	83 (25)	298 (40)	2.09 (1.51-2.88)*	67 (17)	145 (24)	1.47 (1.04-2.07)*
**Lemonade** (≥4 times per week)	53 (16)	60 (8)	0.42 (0.27-0.64)*	91 (23)	103 (17)	0.67 (0.48-0.94)*
**Sugar-sweetened soft drinks** (≥4 times per week)	205 (62)	327 (44)	0.47 (0.35-0.64)*	312 (79)	472 (78)	0.92 (0.66-1.29)
**Diet soft drinks** (≥4 times per week)	96 (29)	208 (28)	1.01 (0.73-1.39)	103 (26)	145 (24)	0.94 (0.68-1.29)
**Candy** (≥4 times per week)	238 (72)	506 (68)	0.77 (0.56-1.06)	257 (65)	375 (62)	0.94 (0.70-1.26)
**Salty snacks** (≥4 times per week)	192 (58)	365 (49)	0.63 (0.47-0.85)*	237 (60)	363 (60)	0.97 (0.73-1.29)

^a^A mean school grade between 0 and 3 was characterized as low, whereas a mean school grade between 4 and 6 was characterized as high.

^b^Adjusted for BMI and maternal and paternal education.

*p < 0.05.

Table 3 presents the physical activity level and use of active commuting relative to academic achievement, with the adjusted analyses demonstrating increased odds of high academic achievement in girls and boys who had a high leisure time physical activity level (AOR: 1.51 (1.10-2.08) and AOR: 1.39 (1.05-1.85), respectively). Similarly, a high academic achievement was associated with active commuting to school among girls (AOR: 1.51 (1.10-2.08)) and boys (AOR: 1.72 (1.26-2.35)).

**Table 3:** **Adjusted odds ratio (AOR) and 95% CI for high academic achievement in relation to physical activity in girls and boys**

	GIRLS			BOYS		
	Low academic achievement ^a^ n%	High academic achievement ^a^ n (%)	AOR ^b^(95% CI)	Low academic achievement ^a^ n (%)	High academic achievement ^a^ n (%)	AOR ^b^(95% CI)
**High leisure time physical activity** ^**c**^	89 (27)	268 (36)	1.51 (1.10-2.08)*	162 (41)	303 (50)	1.39 (1.05-1.85)*
**Active commuting to school** ^**d**^	83 (25)	253 (34)	1.51 (1.10-2.08)*	95 (24)	212 (35)	1.72 (1.26-2.35)*

^a^A mean school grade between 0 and 3 was characterized as low, whereas a mean school grade between 4 and 6 was characterized as high.

^b^Adjusted for BMI and maternal and paternal education.

^c^Five hours or more of leisure time physical activity per week.

^d^Active commuting (biking, walking, etc.) to school.

*p < 0.05.

Table 4 presents smoking and snuffing relative to academic achievement, with adjusted analyses showing decreased odds of a high academic score among girls and boys reporting to be smokers (AOR: 0.18 (0.12-0.25) and AOR: 0.37 (0.25-0.54), respectively) and snuffers (AOR: 0.25 (0.17-0.37) and AOR: 0.51 (0.36-0.72), respectively).

**Table 4:** **Adjusted odds ratio (OR) and 95% CI for high academic achievement in relation to smoking and snuffing in girls and boys**

	GIRLS			BOYS		
	Low academic achievement ^a^ n (%)	High academic achievement ^a^ n (%)	AOR ^b^(95% CI)	Low academic achievement ^a^ n (%)	High academic achievement ^a^ n (%)	AOR ^b^(95% CI)
**Smoking**	122 (37)	74 (10)	0.18 (0.12-0.25)*	91 (23)	54 (9)	0.37 (0.25-0.54)*
**Snuffing**	86 (26)	60 (8)	0.25 (0.17-0.37)*	103 (26)	91 (15)	0.51 (0.36-0.72)*

^a^A mean school grade between 0 and 3 was characterized as low, whereas a mean school grade between 4 and 6 was characterized as high.

^b^Adjusted for BMI and maternal and paternal education.

*p < 0.05.

## Discussion

Few other studies targeting adolescents have reported a very high participation rate similar to the present study, and to the best of our knowledge, no other studies have examined the association between such a wide range of health behaviors and academic achievement among adolescent boys and girls.

In the present study, having a regular intake of breakfast was strongly associated with increased odds of high academic achievement in Norwegian adolescents, particularly in girls. This result is in accordance with previously published studies, which have reported that habitual breakfast consumption is more beneficial than skipping breakfast with regard to cognitive performance and academic achievement in both children and adolescents [7, 8, 21]. Moreover, in terms of providing a greater variety of food groups and adequate energy, a review by Adolphus and coworkers [7] suggested that the quality of habitual breakfast was positively related to academic achievement in children and adolescents.

While other studies focusing on the relationship between meal pattern and academic achievement have primarily focused on the importance of breakfast, our results further emphasize the importance of a regular meal pattern throughout the day. A regular intake of lunch was associated with increased odds of high academic achievement in both boys and girls, and a regular intake of dinner and a regular meal pattern in general were both associated with increased odds of high academic achievement in boys in the present study. Similar to our results, a study among Korean children reported a positive association between the consumption of regular meals, including breakfast, lunch and dinner, and academic achievement [9]. This latter study reported that a regular intake of breakfast and lunch was more important in grades 5 and 8 among girls and boys, whereas a regular intake of dinner was more related to academic achievement in grade 11 among girls [9]. To the best of our knowledge, no other studies have examined the relationship between meal pattern in general and academic achievement among adolescent boys and girls. Additionally, few studies have examined the relationship between specific food intake and academic achievement in adolescent girls and boys. Results from the present study showed increased odds of high academic achievement in girls and boys who had a high intake of fruit and berries and girls having a high intake of vegetables. In contrast, the results exhibited decreased odds of high academic achievement in girls and boys reporting a high intake of lemonade and girls reporting a high intake of sugar- sweetened soft drinks and salty snacks. A study among Spanish adolescents also showed increased odds of a high academic score among girls, but not boys, who had two servings of fruit a day or more [15]. Furthermore, a study among 16,188 American adolescents demonstrated a significant association between the consumption of sugar- sweetened soda and poor academic score [22], while a recent study of first-year Belgian university students reported that a low academic achievement was associated with a high consumption of soda, French fries and alcohol [23]. In addition, a high intake of sweets, salty snacks and fast foods were inversely related, whereas a high intake of fruit and vegetables was positively associated with a high academic achievement in adolescents in Iceland [24, 25]. A study among Norwegian children also concluded that a low intake of fruits and a high intake of sugar-sweetened soft drinks, snacks and fast food was related to having mathematical difficulties in school [10], whereas a comprehensive study among Canadian children has shown that a high diet quality was positively associated with a high academic achievement [11].

In contrast to our study, few other studies have focused on both leisure time physical activity level and active commuting as measures of physical activity, and the association with academic achievement. The results for this study showed a positive association between academic achievement and both leisure time physical activity level and active commuting to school in both boys and girls. Three studies among adolescents in Iceland have also revealed a positive association between physical activity and both school contentment and academic achievement [24–26]. In addition, a Spanish study reported that a high academic score was positively associated with physical activity in adolescent girls, but not in boys [15]. Active commuting to school has also been associated with a better cognitive performance in Spanish girls, but not in boys [17]. On the other hand, a study among Danish adolescents reported that active commuting was positively associated with academic achievement in boys [27]. These gender-specific effects might be explained in part by differences in the mode and duration of physical activity among boys and girls in different countries [15, 28, 29].

The results from the present study also showed a strong inverse relationship between smoking and snuffing and academic achievement in both girls and boys. To the best of our knowledge, very few other studies have explored the relationship between smoking and academic achievement, and no other study has examined the relationship between snuffing and academic achievement in adolescents. Nonetheless, the results from a Canadian study suggested that adolescents who attain high levels of academic achievement are less likely to smoke [19], and a recent study also suggested that smoking both predicts and is predicted by lower academic achievement [30].

Previously published results from this study have also shown a significant association between short time in bed and academic achievement among adolescent girls and boys [31].

Moreover, other underlying characteristics may partially explain why adolescents with healthy lifestyle habits are more likely to succeed academically. Factors such as self-esteem, depressive mood and school contentment have been suggested as mediating factors between the indicators of health behavior and academic achievement [24–26]. A recently published study reported the mediating effect of academic self-efficacy in the relation between school psychological climate and academic achievement in Norwegian adolescents [32]. Furthermore, a longitudinal study has shown that self-discipline was an important predictor of academic achievement in American adolescents [33]. Results from these latter mentioned studies underline the need to further investigate possible mediators of the association between lifestyle habits and academic achievement.

### Strengths and limitations

Because adolescents from families with a low socio-economic status (SES) are more likely to consume a low-quality diet, SES may act as an achievement buffer, thereby partially explaining the relationship between meal pattern, diet and academic achievement [7, 11, 34]. Hence, it is important to account for these variables in cognitive or academic achievement studies, as we have done by adjustment for parental education in the present study. Our study design is also characterized by other strengths, including an exceptionally high participation rate (98.7%). In addition, inter-rated reliability was assessed for all variables included in the study, and the results showed high reliability scores. Lastly, few other studies have explored the relationship between such a wide range of health behaviors and academic achievement in this age group. One limitation of this study is that all the data were self-reported, while another is the cross-sectional design, which does not allow for causal inference. Therefore, the results from this study cannot illuminate the mechanisms that link lifestyle habits and academic achievement.

## Conclusion

In accordance with our hypothesis, increased odds of high academic achievement was shown among adolescents having a regular meal pattern, particularly breakfast and lunch, a high intake of healthy food items and being physically active. Conversely, decreased odds of high academic achievement were shown among adolescents having a high intake of unhealthy food items, and those reporting to be smokers or snuffers. The results from this study have provided important information about the association between a broad range of lifestyle factors and academic achievement in adolescent girls and boys indicating that focus on healthy dietary- and physical activity habits and prevention from smoking and snuffing is of high importance for academic achievement among this target group. Based on these and previous findings, future intervention studies should investigate the combined effect of promoting healthy lifestyle habits and mental health on academic achievement.
